# Acute tubulointerstitial nephritis and IgM deposits on glomerular capillary walls after immunotherapy with nivolumab for metastatic renal cell carcinoma

**DOI:** 10.1007/s13730-019-00424-1

**Published:** 2019-10-11

**Authors:** Taisuke Irifuku, Ayaka Satoh, Hiroki Tani, Kouichi Mandai, Takao Masaki

**Affiliations:** 1 Department of Nephrology, National Hospital Organization Higashihiroshima Medical Center, Higashi-Hiroshima, Japan; 2 Department of Artificial Organs, Akane-Foundation, Tsuchiya General Hospital, Hiroshima, Japan; 3 Department of Diagnostic Pathology, National Hospital Organization Higashihiroshima Medical Center, Higashi-Hiroshima, Japan; 4 Department of Nephrology, Hiroshima University Hospital, Hiroshima, Japan

**Keywords:** Anti-programmed cell death-1 antibody, Acute tubulointerstitial nephritis, Immune-mediated glomerulonephropathy

## Abstract

Nivolumab is an anti-programmed cell death-1 antibody that is utilized as an immune checkpoint inhibitor for several malignancies. However, this agent is associated with immune-related adverse events (irAEs), mainly in the spectrum of autoimmune disease including interstitial pneumonia, colitis, type 1 diabetes, and renal impairment. We herein present the case of a 59-year-old man with renal cell carcinoma who developed worsening renal function approximately 4 months after initiation of nivolumab. Urinalysis showed proteinuria and microscopic hematuria along with increase levels of *N*-acetyl-β-d-glucosaminidase. Renal biopsy revealed acute tubulointerstitial nephritis and thickening of the glomerular basement membranes. Immunofluorescence showed granular IgM deposits in capillary loops. We initiated high-dose prednisolone therapy with nivolumab, which improved renal function and achieved complete remission of proteinuria. Although renal irAEs are considered to be rare and glomerulonephropathy is not typical presentation, physicians need the close monitoring of renal function and urinalysis in patients under immunotherapy with this agents. In addition, our case provides a possible link between nivolumab and immune-mediated glomerulonephropathy.

## Introduction

Immune checkpoint inhibitors (ICIs) are attracting attention as novel cancer therapeutic agents against multiple cancer species, such as melanoma, non-small-cell lung carcinoma, and renal cell carcinoma [1–3]. These agents are monoclonal antibodies targeting anti-cytotoxic T-lymphocyte-associated protein-4 and anti-programmed death-1 (PD-1) signaling pathways. Nivolumab (anti-PD-1 antibody) is considered to enhance tumor-directed immune response by reactivation of cytotoxic T cell, leading to tumor cell lysis [4].

On the other hand, ICIs have been associated with numerous unique side effects, termed immune-related adverse events (irAEs). IrAEs occur in up to 60% of treated patients, usually mild to moderate in grade. The pathophysiology of irAEs is possibly mediated through non-specific immune activation against self-antigens [5]. The most commonly reported irAEs include skin rash, colitis, hepatitis, hypophysitis, interstitial pneumonia, and type 1 diabetes [5]. According to previous reports, renal irAEs are less frequent than other organ involvement [6]. Furthermore, most cases of renal irAEs present as acute tubulointerstitial nephritis; however, IgM deposition on glomerular capillary walls triggered by nivolumab has not been previously reported [7].

Herein, we present the case of a patient with metastatic clear cell renal cell carcinoma treated with nivolumab, who developed acute tubulointerstitial nephritis and immune-mediated glomerulonephropathy.

## Case report

A 59-year-old Japanese man was referred and admitted to our department owing to progressive deterioration of renal function and new onset proteinuria after immunotherapy with nivolumab.

**Fig. 1:**
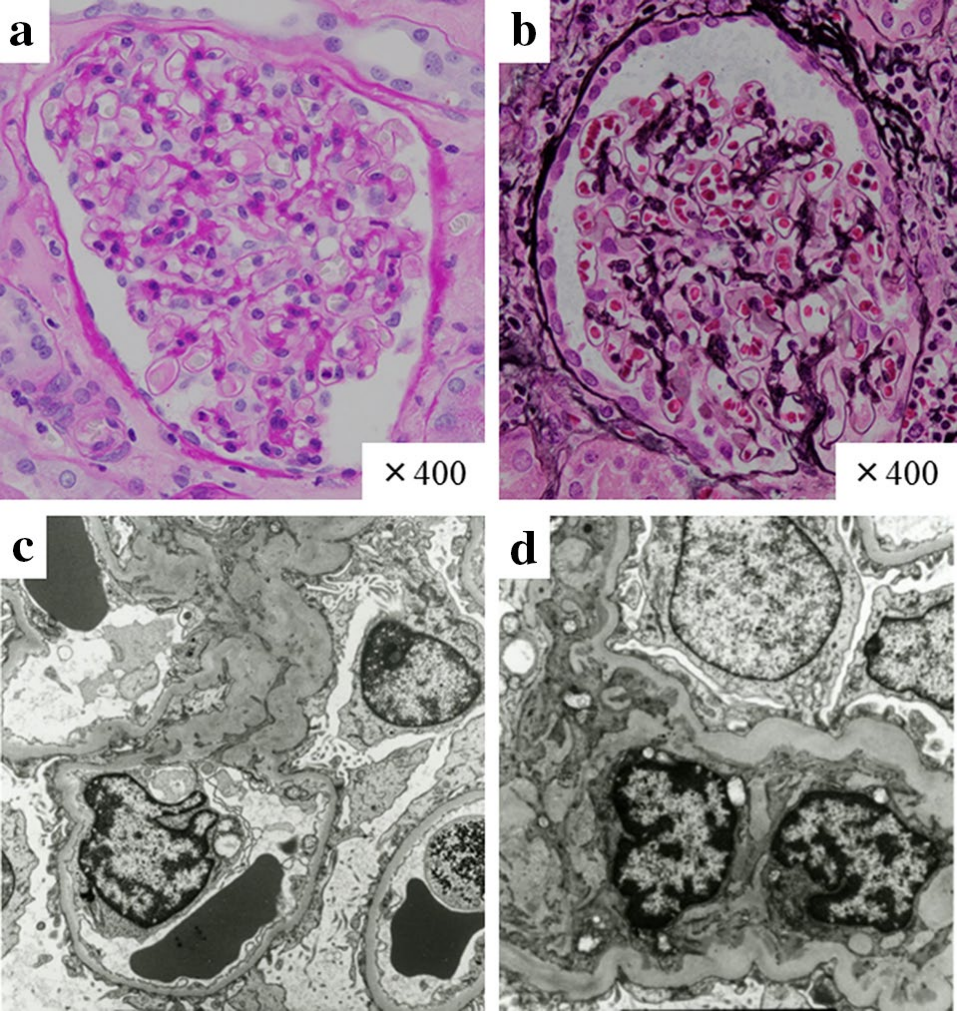
Histological findings on nephrectomy specimens. Light microscopy revealed minor glomerular abnormality (**a**, **b**). Electron microscopy showed extensive foot process effacement without any immune complex deposits (**c**, **d**)

With regard to the patient’s history, he was referred to our medical center with kidney tumor and nephrotic syndrome 4 years previously. Left nephrectomy was performed for treatment of renal cell carcinoma (cT2N2M0) diagnosed using positron emission tomography–computed tomography. Histopathological analysis of the nephrectomy specimens confirmed clear cell renal cell carcinoma of the left kidney and minor glomerular abnormalities (Fig. 1). Immunofluorescence study demonstrated absence of deposits of immunoglobulins and complements. Electron microscopy showed extensive foot process effacement without any immune complex deposits (Fig. 1). Surgical resection of the tumor resulted in complete remission of proteinuria without corticosteroid therapy. After operation, the patient received several adjuvant therapies, including sunitinib, sorafenib, and pazopanib. However, 3 years after the initial diagnosis, the cancer progressed to stage IV (T0N2M1) with multiple metastases to the bone, adrenal grand, and spleen. He was then started on biweekly treatments with nivolumab (3 mg/kg, by intravenous drip infusion). Although nivolumab was effective, an acute increase in serum creatinine (Cre) levels (from 1.13 to 2.39 mg/dL) was observed approximately 4 months after initiation of nivolumab. His medications were rosuvastatin for dyslipidemia, esomeprazole for gastroesophageal reflux disease, and pregabalin for chronic pain.

On admission, his height was 172 cm, body weight was 60.9 kg, blood pressure was 123/84 mmHg, and pulse rate was 93/min. Physical examination revealed no abnormal clinical signs, such as ophthalmologic findings suggesting uveitis. Urinalysis showed proteinuria (1.5 g/day), mild hematuria (10–19/HPF), and a few granular casts. *N*-acetyl-β-d-glucosaminidase activity and β-2 microglobulin were elevated at 36.3 U/L and 7754 μg/L, respectively. The results of laboratory blood tests were as follows: white blood cell count 9900/μL, with 71.6% neutrophils, 2.4% eosinophils, hemoglobin 12.1 g/dL, platelet count 26.2 × 10^4^/μL, serum albumin 3.7 g/dL, serum Cre 3.09 mg/dL, blood urea nitrogen 35.8 mg/dL, C-reactive protein 1.9 mg/dL, IgG4 33.5 mg/dL (0.9%), and angiotensin-converting enzyme 9.6 U/L. Furthermore, tests were negative for anti-nuclear, anti-neutrophil cytoplasmic, anti-Sjogren syndrome (SS)-A, and anti-SS-B antibodies (Table 1).

**Table 1:** Laboratory data at renal biopsy

*Urinalysis*		*Chemistry*		*Serology*	
Specific gravity	> 1.030	T.Pro	8.5 g/dL	IgG	3651 mg/dL
pH	5.5	Alb	3.7 g/dL	IgA	226 mg/dL
Protein	3 +	AST	20 IU/L	IgM	146 mg/dL
Occult blood	3 +	ALT	13 IU/L	C3	177 mg/dL
Protein	1.5 g/day	LDH	180 IU/L	C4	29 mg/dL
NAG	26.3 IU/L	ChE	263 IU/L	ANA	Negative
β2-MG	7754 Hg/L	yGTP	36 IU/L	MPO-ANCA	Negative
RBC	20–29/HPF	CK	41 IU/L	PR3-ANCA	Negative
WBC	1–4/HPF	UA	6.9 mg/dL	SS-A Ab	Negative
Granular cast	30–49/WF	BUN	35.8 mg/dL	SS-B Ab	Negative
		Cre	2.39 mg/dL	ACE	9.6 IU/L
*CBC*		Na	138 mEq/L	IgG4	33.5 mg/dL
WBC	9900/μL	K	4 mEq/L	HBs Ag	Negative
Neutro	71.6%	CI	107 mEq/L	HCV Ab	Negative
Lympho	18.9%	Ca	9.1 mg/dL		
Eosino	2.4%	IP	3 mg/dL		
RBC	450 × 10^4^/μL	Mg	1.5 mg/dL		
Hb	12.1 g/dL	Tchol	146 mg/dL		
Ht	31%	TG	118 mg/dL		
Pit	26.2 × 10^4^/uL	LDL-C	70 mg/dL		
		HDL-C	53 mg/dL		
*Blood coagulation test*		Glu	122 mg/dL		
PT	99%	CRP	1.92 mg/dL		
PT-INR	1.01				
APTT	34 s				

**Fig. 2:**
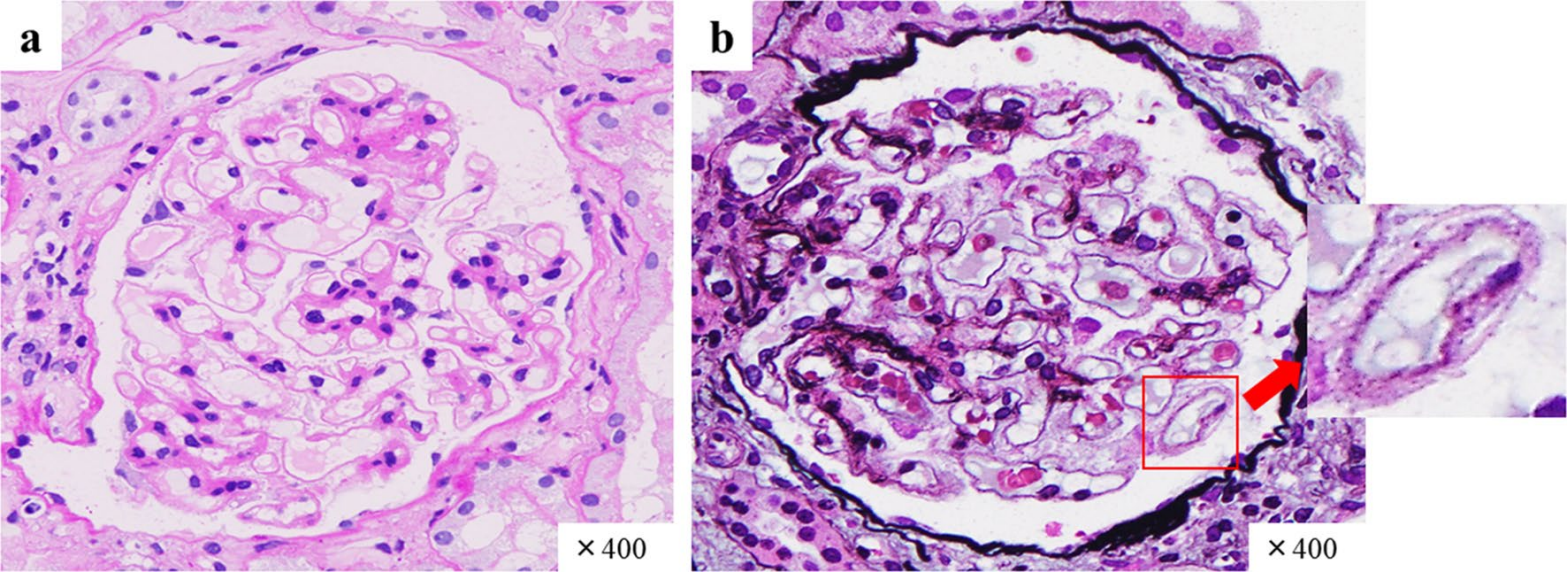
Periodic acid–Schiff staining and Periodic Acid–Methenamine Silver stain demonstrated thickening of glomerular basement membranes **a** with a bubble-like appearance (**b**; red arrow)

**Fig. 3:**
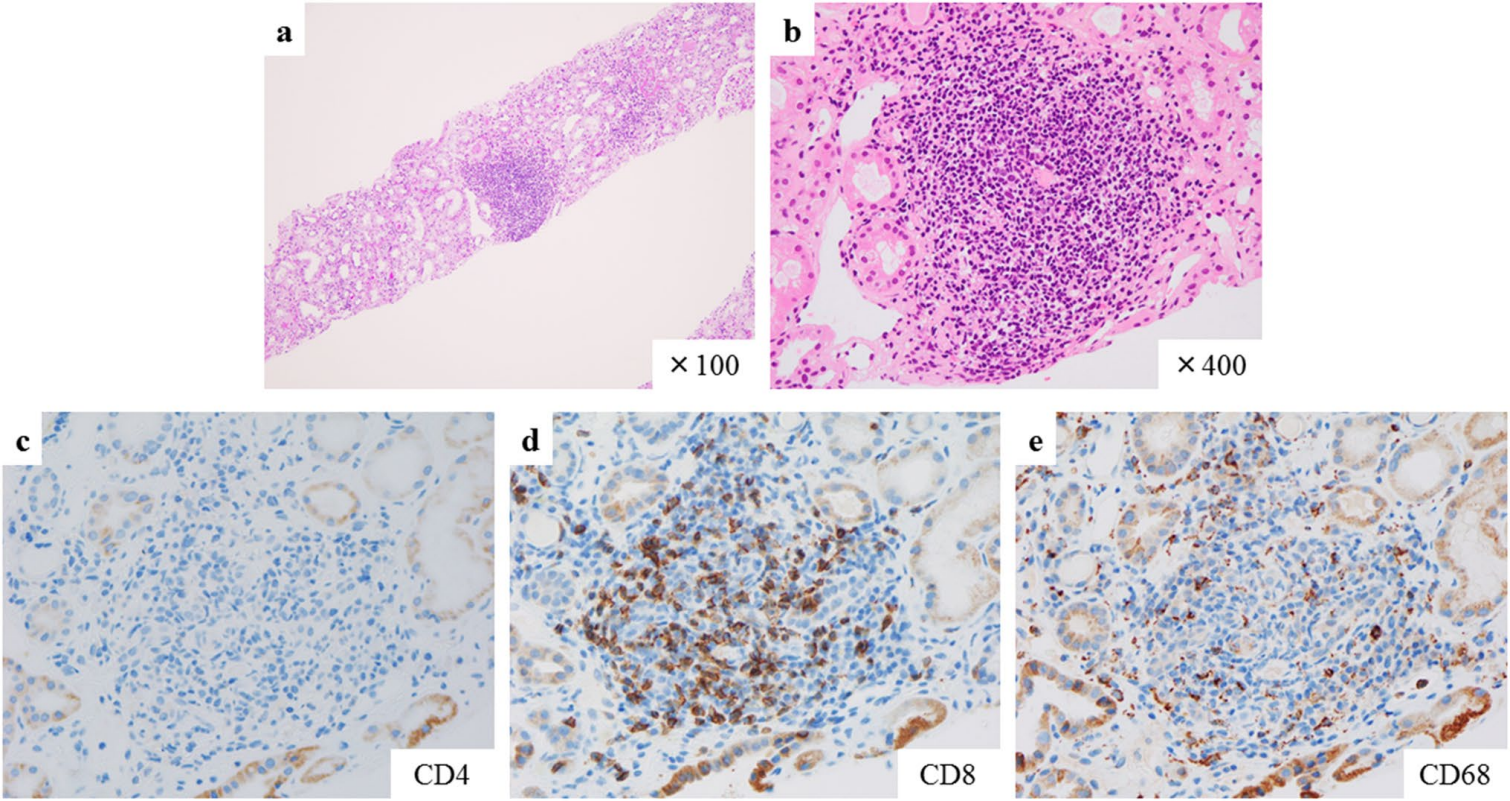
Hematoxylin and eosin staining showed focal interstitial inflammatory cell infiltration (**a**, **b**). Immunohistochemical staining for CD4 (**c**), CD8 (**d**), and CD68 (**e**). The marked infiltration of CD8^+^ and CD68^+^ cells is observed within the lymphoid follicles

**Fig. 4:**
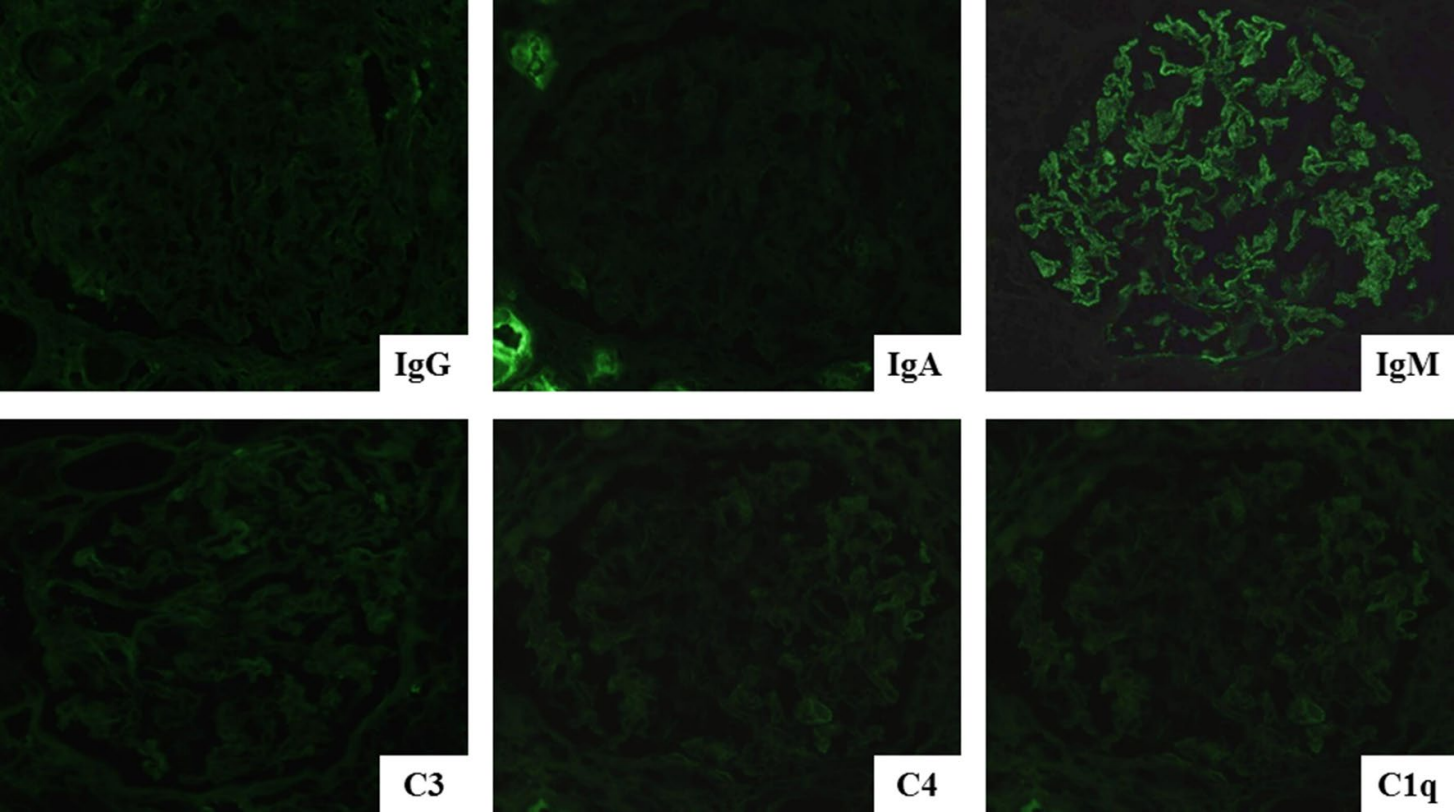
Immunofluorescence revealed only IgM deposition in capillary loops

**Fig. 5:**
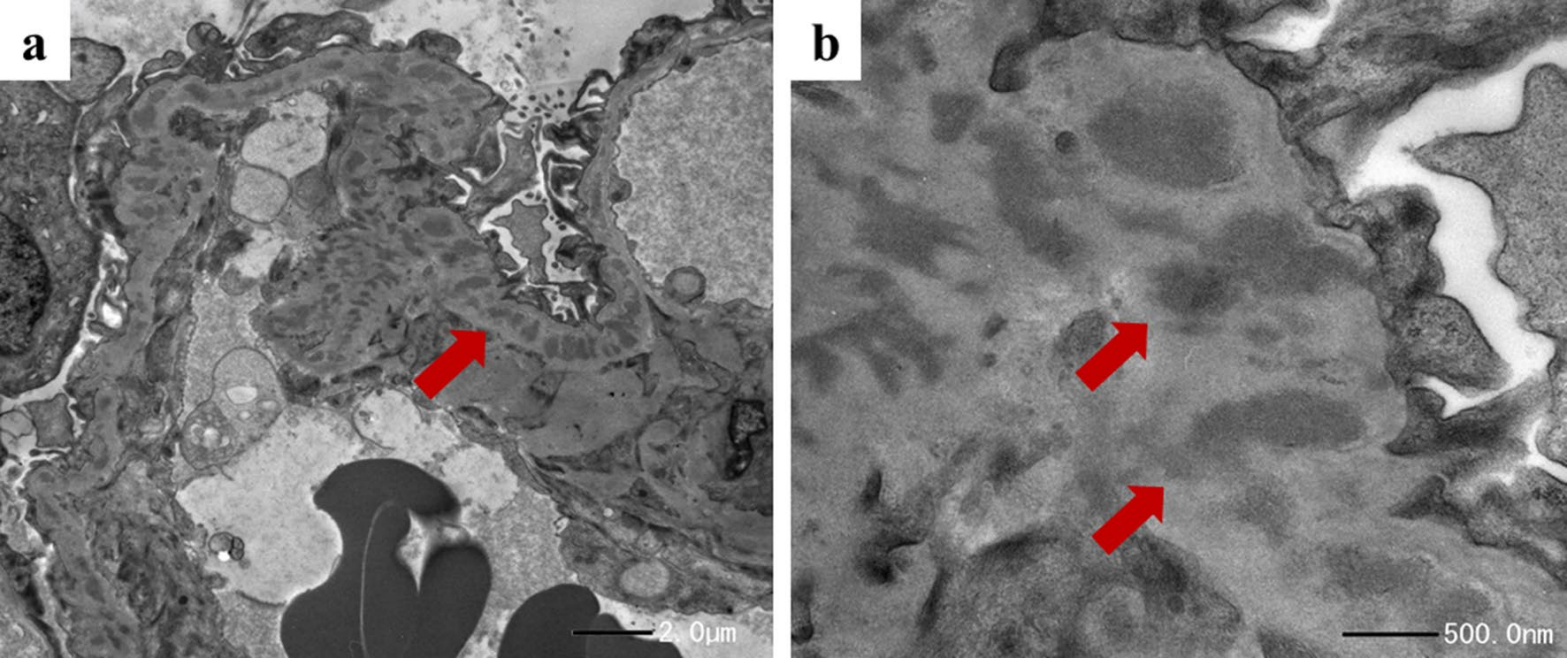
Electron microscopy showed intra-membranous electron-dense deposits (arrow) in glomerular capillary wall, but not in subendothelial or mesangial area

**Fig. 6:**
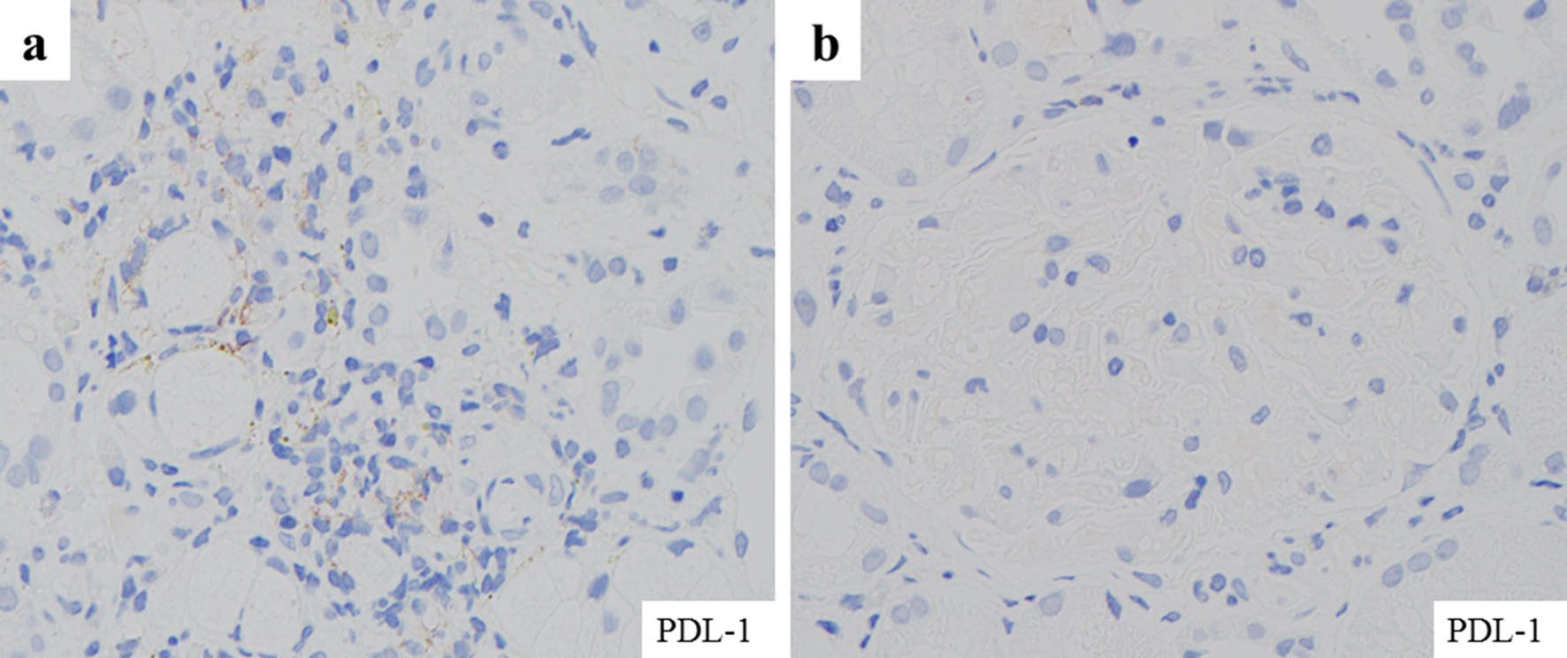
Immunohistochemical staining demonstrated the slightly PDL-1 expression in tubular epithelial cells but not in glomeruli

Renal biopsy was immediately performed to clarify the cause of worsening renal function. It contained total 17 glomeruli, of which 4 demonstrated global sclerosis. Light microscopy revealed thickening of the glomerular basement membranes with a bubble-like appearance, tubulitis, and focal interstitial inflammatory cell infiltration (Figs. 2, 3). The infiltrates were primarily composed of CD8^+^ T cells and CD68^+^ macrophages with admixed eosinophils and neutrophils (Fig. 3). Immunofluorescence staining showed only granular IgM deposition in the capillary loops. Staining for IgG, IgA, C3, C4, and C1q was negative (Fig. 4). Electron microscopy showed intra-membranous electron-dense deposits (arrow) in glomerular capillary wall, but not in subendothelial or mesangial area (Fig. 5). In addition, immunohistochemical staining revealed the slightly PDL-1 expression in tubular epithelial cells but not in glomeruli (Fig. 6). Based on these findings, we considered this patient as having acute tubulointerstitial nephritis and immune-mediated glomerulonephropathy.

**Fig. 7:**
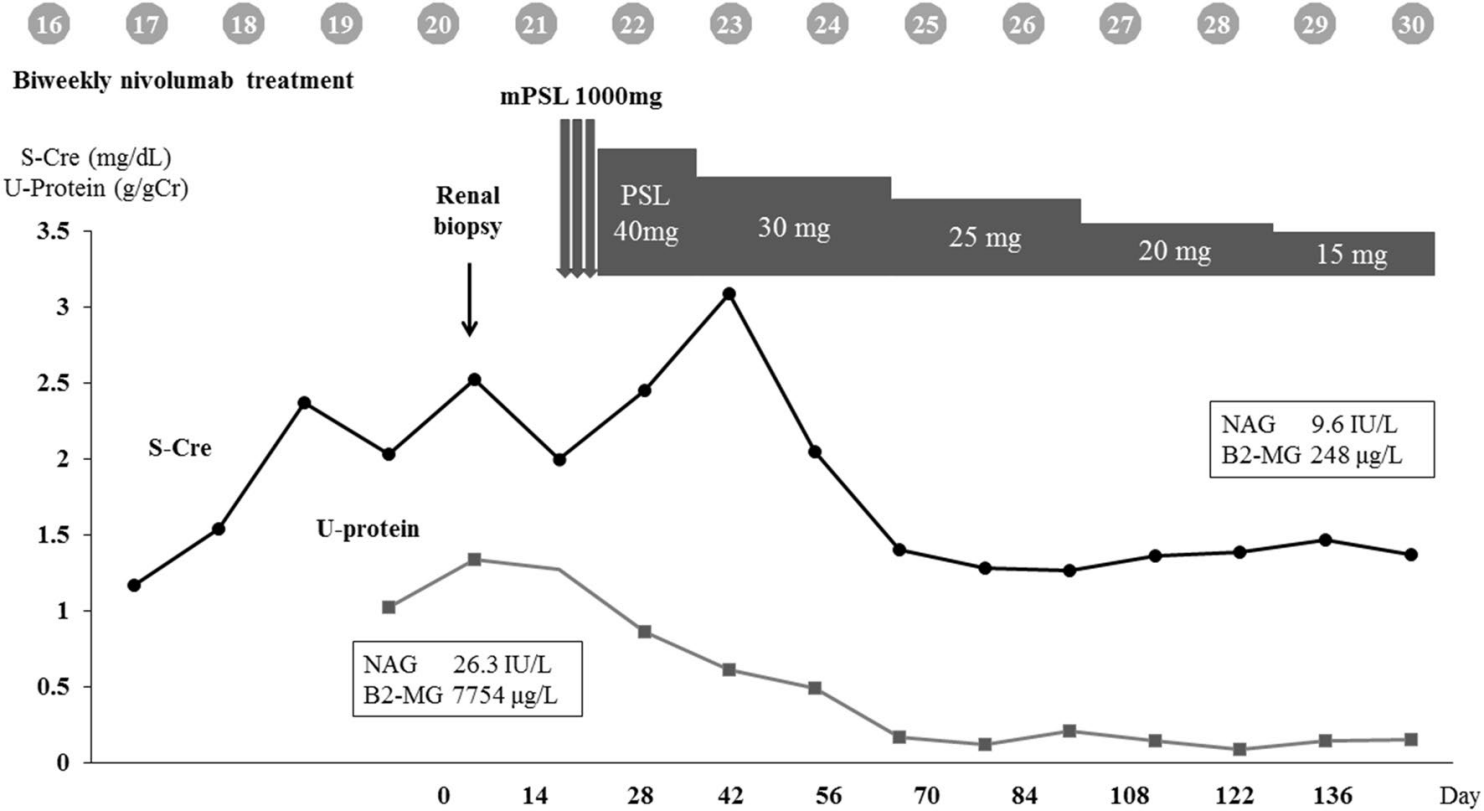
Summarized clinical course. *S-Cre* serum creatinine, *PSL* prednisolone, *NAG N*-acetyl-β-d-glucosaminidase activity, *β2-MG* β-2 microglobulin

After biopsy-proven diagnosis, steroid therapy was initiated with intravenous methylprednisolone 1 g/day for 3 days, followed by oral prednisolone at 40 mg/day. Although a majority of the previous reports suggested that immunotherapy should be withdrawn when there was the possibility of irAEs, we continued nivolumab by considering the survival benefit. Steroid therapy led to rapid improvement in his renal function (serum Cre 1.32 mg/dL) along with complete remission of urinary protein excretion (0.17 g/gCre), thus allowing him to continue with nivolumab therapy. The summarized clinical course is shown in Fig. 7.

## Discussion

This case report highlights that ICIs could induce not only acute tubulointerstitial nephritis, but also immune-mediated glomerulonephropathy as renal irAEs. In a previous report, two different types of ICI-related kidney injury have been reported: acute tubulointerstitial nephritis and glomerular disease [8]. Most cases of renal irAEs present as acute tubulointerstitial nephritis [9]. In the largest case series of renal irAEs [7], subepithelial and intra-membranous deposits were observed in only one patient treated with ipilimumab. As shown in Fig. 6, PDL-1 was observed in tubular epithelial cells but not in glomeruli. This finding suggests that interstitial nephritis occurred locally within the kidneys triggered by nivolumab, whereas glomerulonephropathy developed as a result of the systemic formation of immune complexes following the administration of nivolumab. To the best of our knowledge, this is the first report of IgM deposits on glomerular capillary wall following nivolumab treatment confirmed on biopsy. Similar cases would increase henceforth, and the accumulation of cases is important for elucidation of underlying mechanism and proper management of renal irAEs.

The progress of ICIs has revolutionized the therapy for variety of cancers, and ICIs have been approved for a number of types of cancers. ICIs enhance anti-tumor immunity by blocking co-inhibitory molecules that are expressed on both T cells and tumor cells [10]. The PD-1-blocking antibody “nivolumab” is approved by the Food and Drug Administration for the treatment of metastatic melanoma, non-small cell lung cancer, classical Hodgkin’s lymphoma, and renal cell carcinoma [11]. The incidence of renal adverse events was reported to be rare (< 1%) in randomized control trials of nivolumab [12], where the subjects were administered 3 mg/kg nivolumab every 2 weeks, similar to that in our case [12]. However, recent studies have reported that the incidence of renal toxicities might be higher than that previously reported [9]. Furthermore, these patients developed acute kidney injury without any symptoms, and pyuria was the only abnormality that was frequently observed in urinalyses. Therefore, any deterioration in renal function or abnormalities seen on urinalysis should raise a suspicion of ICI-associated nephrotoxicity, especially in the absence of background therapy with other agents that may worsen renal function. Early recognition of these renal irAEs by treating oncologists may be most important for the subsequent clinical course and recovery of renal function.

The patient had minimal change nephrotic syndrome when he underwent nephrectomy for renal cell carcinoma 4 years ago. Minimal change nephrotic syndrome is common among nephrotic syndromes found in patients with malignant lymphoma, whereas there is no evidence to suggest that renal cell carcinoma is associated with minimal change nephrotic syndrome. In this case, intra-membranous deposits may have appeared along with renal cell carcinoma; however, such findings were not observed in the resected specimen of the left kidney.

An animal study demonstrated that PD-1 knockout mice developed lupus-like glomerulonephritis with predominant IgG3 deposition [13]. This finding suggests that PD-1 signaling pathway is involved in immune-mediated renal inflammation. Although we found 41 cases of nephropathy in which IgM deposition was found in previous reports [14], these reports described mesangial deposition of IgM, which is different from our case. In addition, none of the studies performed experiments using animal models or described the possible mechanisms of IgM deposition. Although it may be difficult to prove the causal relationship between ICIs use and IgM deposits on glomerular capillary wall, we considered that nivolumab played an important role in the pathogenesis of immune-mediated glomerulonephropathy.

Steroid therapy is becoming a standard treatment in patients with acute tubulointerstitial nephritis as renal irAEs [15]. In the aforementioned case series of Cotazar et al. [7], complete or partial remission of renal damage was observed in 9 out of 10 patients with acute tubulointerstitial nephritis who received short-term steroid therapy; in contrast 2 patients who were not administered corticosteroids showed deterioration in renal function. Although the implicated ICIs were discontinued in previously reported patients, our patient was continued on biweekly treatment with nivolumab. As other anti-tumor agents were not effective so far, we emphasized the survival benefit by continuing the nivolumab therapy. At the time of writing, we could arrest the progression of cancer, and his renal function and proteinuria were maintained at baseline levels. Our case provides the possibility that the combination of corticosteroid therapy allows us to continue with the ICIs even when renal irAEs occurred.

In summary, we reported a newly diagnosed case of acute tubulointerstitial nephritis and immune-mediated glomerulonephropathy following immunotherapy with nivolumab. Because ICIs will be used more widely, careful monitoring of the renal function and proteinuria, as well as timely consideration of biopsy of the kidney is important.
